# Loss of tumor suppressor Merlin results in aberrant activation of Wnt/β-catenin signaling in cancer

**DOI:** 10.18632/oncotarget.7494

**Published:** 2016-02-19

**Authors:** K. Adam Morrow, Shamik Das, Erhong Meng, Mitchell E. Menezes, Sarah K. Bailey, Brandon J. Metge, Donald J. Buchsbaum, Rajeev S. Samant, Lalita A. Shevde

**Affiliations:** ^1^ Mitchell Cancer Institute, University of South Alabama, Mobile, AL, USA; ^2^ Department of Pathology, University of Alabama at Birmingham, Birmingham, AL, USA; ^3^ Department of Radiation Oncology, University of Alabama at Birmingham, Birmingham, AL, USA; ^4^ Comprehensive Cancer Center, University of Alabama at Birmingham, Birmingham, AL, USA

**Keywords:** NF2, Merlin, breast cancer, Wnt, β-catenin

## Abstract

The expression of the tumor suppressor Merlin is compromised in nervous system malignancies due to genomic aberrations. We demonstrated for the first time, that in breast cancer, Merlin protein expression is lost due to proteasome-mediated elimination. Immunohistochemical analysis of tumor tissues from patients with metastatic breast cancer revealed characteristically reduced Merlin expression. Importantly, we identified a functional role for Merlin in impeding breast tumor xenograft growth and reducing invasive characteristics. We sought to determine a possible mechanism by which Merlin accomplishes this reduction in malignant activity. We observed that breast and pancreatic cancer cells with loss of Merlin show an aberrant increase in the activity of β-catenin concomitant with nuclear localization of β-catenin. We discovered that Merlin physically interacts with β-catenin, alters the sub-cellular localization of β-catenin, and significantly reduces the protein levels of β-catenin by targeting it for degradation through the upregulation of Axin1. Consequently, restoration of Merlin inhibited β-catenin-mediated transcriptional activity in breast and pancreatic cancer cells. We also present evidence that loss of Merlin sensitizes tumor cells to inhibition by compounds that target β-catenin-mediated activity. Thus, this study provides compelling evidence that Merlin reduces the malignant activity of pancreatic and breast cancer, in part by suppressing the Wnt/β-catenin pathway. Given the potent role of Wnt/β-catenin signaling in breast and pancreatic cancer and the flurry of activity to test β-catenin inhibitors in the clinic, our findings are opportune and provide evidence for Merlin in restraining aberrant activation of Wnt/β-catenin signaling.

## INTRODUCTION

Merlin, also known as schwannomin or neurofibromin 2, was first discovered in 1993 as the protein encoded by the *NF2* gene [1, 2]. Merlin is a member of the Band 4.1 family of cytoskeletal linker proteins that include the ERM (Ezrin, Radixin, Moesin) proteins [3]. Traditionally, proteins of this family process signals from the extracellular matrix and transmit them to proteins inside the cell. Loss of heterozygosity of the *NF2* gene or mutations within the gene manifests frequently as neurofibromatosis 2, schwannomas, meningiomas, and ependymomas. Mutations of the *NF2* gene are also found in mesotheliomas [4-6], prostate cancer [7], colorectal cancer [8], melanoma, and thyroid cancer [9]. This suggests Merlin may function to suppress tumor growth and progression in a variety of tissues.

Three independent studies failed to identify mutations of the *NF2* gene in breast cancers [10-12]. Concordant with this, we observed that in breast cancer there was no significant change in Merlin transcript levels. However, we detected a significant loss of Merlin protein expression in early and advanced breast tumor tissues, in particular, those cases with metastases [13]. We identified the role of growth factor signaling in causing degradation of Merlin protein, thereby reducing the cellular Merlin pool in breast cancer cells. Several studies have revealed the ability of Merlin to negatively regulate cell growth and proliferation [14-16]. Merlin can reduce cell proliferation by binding to the cytoplasmic tail of the CD44 receptor. This binding inhibits the interaction of Hyaluronic Acid (HA) with CD44 and suppresses downstream signaling events [15, 17]. Merlin also inhibits cell cycle progression through suppression of PAK1-mediated expression of cyclin D1 [18]. We have shown that Merlin inhibited anchorage-dependent growth, promoted contact inhibition of breast cancer cells, and mitigated their ability to grow as xenografts. Merlin also reduced invasion and motility of metastatic breast cancer cells [13].

Detachment of cells involves profound rearrangements of structural molecules characterized by disruption of the cadherin-catenin complex with the cytoskeleton. β-catenin is an integral member of adherens junctions that regulates the cellular dynamics of cell attachment or detachment [19]. β-catenin has dual roles within the cell - as the main mediator of Wnt signaling and as a junctional protein involved in cell-cell contact. At the membrane as a member of adherens junctions, β-catenin forms a complex with cadherin and α-catenin. Dissociation of β-catenin from cadherin elevates levels of nuclear β-catenin, a frequent occurrence in tumorigenesis [20]. Cytoplasmic and/or nuclear β-catenin affords a poor prognosis in several cancer types including colorectal cancer [21], breast cancer [22], lung cancer [23], and hepatocellular carcinoma [24]. Axin is a scaffolding protein that functions in multiple signaling cascades including Wnt, p53, and transforming growth factor β (TGFβ) pathways [25, 26]. Upon phosphorylation, Axin undergoes a conformational change and its affinity for GSK-3β is enhanced [27], leading to a more active destruction complex and thus inhibiting Wnt signaling. In principle, stabilizing Axin protein could result in decreased β-catenin signaling. This is supported by the fact that Axin overexpression leads to β-catenin degradation even in cells with non-functional APC (Adenomatosis polyposis coli) [28]. It was recently determined that loss of Axin1 expression is a key event in breast cancer progression [29]. As such, stabilization of Axin1 may lead to decreased tumor burden in breast cancer.

In this study we present novel, exciting observations that Merlin binds β-catenin and restricts its nuclear entry. By mediating increased cellular levels of Axin1, Merlin also facilitates proteasomal degradation of β-catenin, thereby reducing the overall pool of β-catenin. Loss of Merlin enables nuclear translocation of β-catenin with a concomitant increase in β-catenin-mediated transcriptional activity in breast and pancreatic carcinoma cells. Importantly, we show that loss of Merlin sensitizes tumor cells to inhibition by compounds that inhibit β-catenin-mediated activity. Thus, our study provides compelling evidence for Merlin in reducing the malignant activity of pancreatic and breast cancer, in part by suppressing the Wnt/β-catenin pathway.

## RESULTS

### Merlin interacts with β-catenin

**Figure 1:**
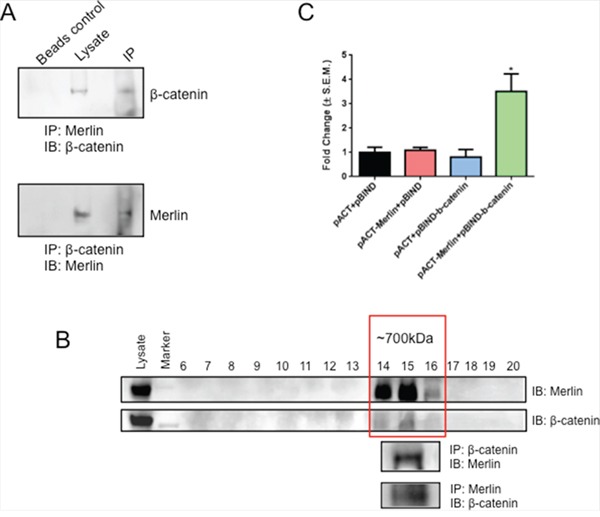
Merlin interacts with β-catenin **A.** Merlin is able to immunoprecipitate β-catenin. Conversely, β-catenin is able to immunoprecipitate Merlin. Cell lysate of SUM159 cells transfected with Merlin was immunoprecipitated for Merlin and immunoblotted with anti-β-catenin antibody. Simultaneously, the cell lysate was immunoprecipitated for β-catenin and immunoblotted with anti-Merlin antibody. **B.** Merlin and β-catenin co-elute and are able to immunoprecipitate one another. MCF10AT cell lysate was subjected to FPLC and the fractions were immunoblotted with anti-Merlin or anti-β-catenin antibodies. Fractions positive for Merlin and β-catenin [14, 15, 16] were pooled and immunoprecipitated for Merlin and immunoblotted with anti-β-catenin antibody. Fractions were also immunoprecipitated for β-catenin and immunoblotted with anti-Merlin antibody. **C.** Merlin interacts with β-catenin in a mammalian two hybrid system. SUM159 cells were co-transfected with pACT-Merlin, pBIND-β-catenin, and pGL4.31. Luciferase activity was measured and normalized to total protein. Error bars represent standard error of the mean (S.E.M.). * indicates statistically significant differences relative to respective control (p=0.02 to 0.03 relative to all other bars).

Given the important role of Merlin in contact inhibition and its role in stabilizing adherens junctions in Schwann cells and mouse embryo fibroblasts (MEFs) [30, 31], we assessed the possible interaction between Merlin and β-catenin in SUM159 cells in light of our earlier data demonstrating the functional relevance of Merlin [13]. As seen in Figure 1A, Merlin co-immunoprecipitated with β-catenin from the lysate of SUM159 cells with stably restored Merlin. This was also confirmed by reverse co-immunoprecipitation with β-catenin in these cells. In order to determine if β-catenin and Merlin form a larger complex, we subjected the lysate of MCF10AT cells to FPLC. MCF10AT cells express endogenous Merlin, thus enabling us to study a physiologically relevant interaction system. Fractions from FPLC ranging in molecular weight from mega Daltons to kilo Daltons were analyzed by immunoblotting for Merlin and β-catenin. Merlin and β-catenin co-eluted in fractions corresponding to ~700KDa molecular weight. These fractions were pooled and evaluated for possible interaction between Merlin and β-catenin by forward and reverse co-immunoprecipitation approaches (Figure 1B). In order to further ascertain the interaction at the molecular level, we conducted a mammalian two-hybrid luciferase assay. We see a robust activation (p < 0.05) of luciferase activity in SUM159 cells transfected with the vectors encoding Merlin and β-catenin, but not the corresponding controls (Figure 1C). Cumulatively, the data supports an interaction between Merlin and β-catenin.

### Merlin alters the activity and sub-cellular localization of β-catenin

**Figure 2:**
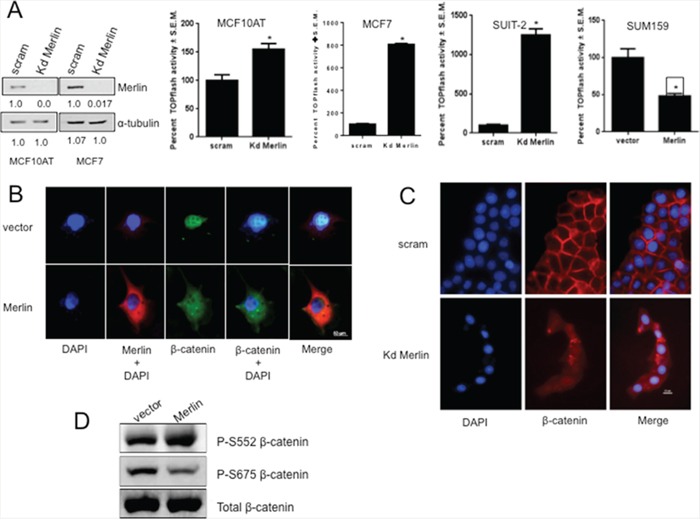
Merlin suppresses β-catenin-mediated transcriptional activity by altering sub-cellular localization of β-catenin **A.** Abrogating Merlin in MCF10AT (kd Merlin; p=0.0018) and MCF7 (p=0.0065) breast cancer cells and SUIT-2 pancreatic cancer cells (p=0.0001) resulted in significant upregulation of TOPflash (TCF/LEF-β-catenin-mediated transcription) activity. Merlin significantly suppressed β-catenin-mediated transcriptional activity in stable transfectants of SUM159 cells (p=0.0147). Each group was assayed in triplicate, repeated at least twice with luciferase readings being normalized to β-galactosidase. * indicates statistically significant differences relative to respective control. The immunoblot shows the extent of knockdown of Merlin expression; the numbers below the blots indicate relative band intensity following densitometry. **B & C.** Merlin alters sub-cellular localization of β-catenin. SUM159 cells (vector control or Merlin-expressing) were stained for Merlin, β-catenin, and mounted in DAPI (Vectashield). MCF7 cells transfected with pSuper scrambled or pSuper-shMerlin Kd Merlin were stained for β-catenin and mounted in DAPI (Vectashield). **D.** Restoration of Merlin results in reduced pS675-β-catenin protein levels in SUM159 cells stably expressing Merlin. Lysates were immunoblotted for total-β-catenin and phospho-β-catenin. Lysates were loaded based on equal total β-catenin expression.

We tested the effect of Merlin on β-catenin-mediated transcriptional activity using TOPFlash activity as a read-out. Abrogating Merlin from MCF10AT and MCF7 breast cancer cells caused a significant (p < 0.05) upregulation of TOPFlash activity indicating activation of β-catenin–mediated transcription (Figure 2A). Interestingly, when we analyzed a panel of pancreatic cancer cell lines, we observed a notable inverse expression between β-catenin and Merlin (Supplementary Data 1A). Silencing Merlin from the MIA PaCa-2 (Supplementary Data 1B) and SUIT-2 (Figure 2A) pancreatic cancer cells significantly (p < 0.05) upregulated TOPFlash activity. MCF7 cells knocked down for Merlin showed an increase in the interaction between endogenous β-catenin and the TCF4 transcription factor (Supplementary Data 1C), supporting the upregulated TOPFlash activity. Conversely, restoration of Merlin in SUM159 (Figure 2A) significantly (p < 0.05) decreased TOPFlash activity suggesting that Merlin inhibits Wnt/β-catenin activity. This inhibition might occur by altered β-catenin subcellular localization, downregulated β-catenin transcript levels, or decreased β-catenin protein levels. Thus, to test the effect of Merlin expression on β-catenin localization, we evaluated SUM159 restored for Merlin. We assessed β-catenin localization in these cells by immunofluorescence. While the image acquisition time for β-catenin was much longer than for Merlin, in contrast to control cells that showed predominantly nuclear β-catenin, Merlin expression resulted in re-localization of β-catenin from the nucleus to a diffuse presence throughout the cell (Figure 2B). We also assessed the impact of silencing endogenous Merlin in MCF7 breast cancer cells. These cells display characteristically membranous β-catenin. Silencing of endogenous Merlin in MCF7 cells resulted in β-catenin that was no longer restricted at the cell membrane (Figure 2C). This also was the case when we silenced endogenous Merlin from MIA PaCa-2 cells (Supplementary Data 1D), suggesting that Merlin is critical for retaining membranous β-catenin. This is supported by the observation that the levels of active β-catenin are increased in MCF7 cells upon silencing endogenous Merlin (Supplementary Data 1E). When β-catenin dissociates from an adherens junction, levels of nuclear β-catenin become elevated. This translocation of β-catenin is a frequent occurrence in tumorigenesis. There are several residues on β-catenin that signal dissociation from the cadherin-catenin complex, resulting in elevated cytoplasmic and nuclear β-catenin. When stimulated with epidermal growth factor (EGF), AKT phosphorylates β-catenin on Serine 552. This phosphorylation, both *in vitro* and *in vivo*, results in enhanced non-membranous β-catenin [32, 33]. Also, upon prostaglandin E (1) and dibutyryl cAMP stimulation, PKA can phosphorylate β-catenin at Serine 675. Phosphorylation at Serine 675 induces β-catenin accumulation in the nucleus and increases β-catenin-mediated transcriptional activity [34, 35]. In agreement with Merlin-induced non-membranous cytoplasmic re-localization of β-catenin in SUM159 cells, phospho-Serine675 β-catenin levels were reduced in Merlin-expressing cells. The phosphorylation status of Ser552 was unaffected (Figure 2D). Cumulatively the data (Figure 2) suggest that Merlin restricts Wnt/β-catenin signaling by decreasing the nuclear pool of β-catenin.

### Loss of Merlin causes upregulation of osteopontin, a bonafide β-catenin transcriptional target gene

**Figure 3:**
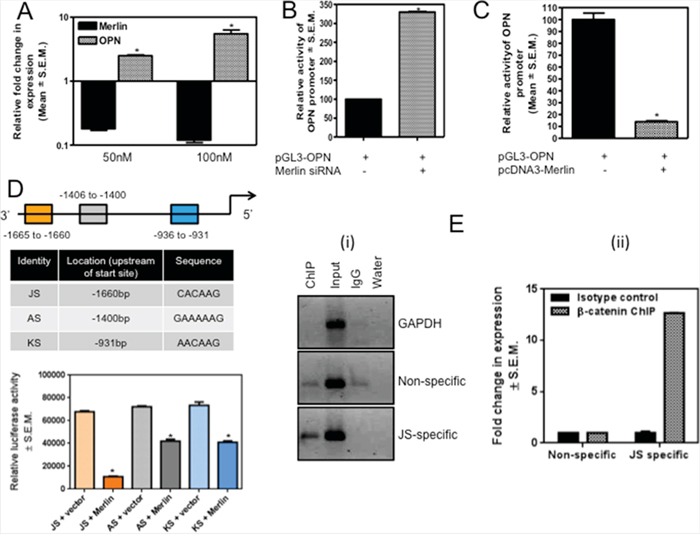
Merlin regulates OPN via modulation of the transcriptional activity of β-catenin **A.** Transfection of Merlin-targeting siRNA (50nM and 100nM) in MCF10AT cells results in a notable (p=0.008) decrease in Merlin with a concordant increase in the levels of endogenous OPN. Merlin and OPN were assessed by real-time quantitative RT-PCR and normalized to endorse control gene, GAPDH. * indicates statistically significant differences relative to respective control. **B.** Silencing endogenous Merlin in MCF10AT cells upregulates OPN promoter activity. Merlin-targeting siRNA was co-transfected with the OPN promoter construct and luciferase activity was measured. Data is normalized to total protein. * indicates statistically significant differences relative to respective control (p=0.004). **C.** Merlin decreases OPN promoter activity. Merlin-expressing construct was co-transfected with OPN promoter in SUM159 cells. Promoter activity was normalized to total protein. * indicates statistically significant differences relative to respective control (p < 0.0001). **D.** The schematic shows the location of the three β-catenin-binding sites in the OPN promoter; the locations of these sites with respect to the transcription start site and the sequences of these sites are indicated in the table. The three reporter constructs, each bearing one of the sites, were co-transfected with a Merlin-expressing construct in SUM159 cells. While Merlin influences β-catenin-mediated suppression of OPN promoter through all three sites (p value for JS=0.0002; p-value for AS=0.0016; p-value for KS=0.0008), Merlin appears to maximally modulate the activity from the JS construct. * indicates statistically significant differences relative to respective control. **E.** ChIP assay ascertains that β-catenin binds the JS site on the OPN promoter. SUM159 genomic DNA was harvested, enzymatically sheared, and immunoprecipitated with non-specific IgG (lane 3) or anti-β-catenin (lane 1) and subjected to PCR analysis. (i) β-catenin binds the JS site within OPN promoter. Input represents sheared chromatin. Non-specific panel represents primers that amplify a region upstream of JS (does not harbor any TCF/LEF binding sites). GAPDH served as a positive control. (ii) The graph represents quantitative PCR of the immunoprecipitated DNA. The pull-down efficiency by JS-specific primers is 12-fold greater than with the non-specific primers.

The osteopontin (OPN) oncoprotein has been reported as one of the transcriptional targets of β-catenin in rodent mammary, bladder, and kidney carcinoma cells [19, 36]. MCF10AT cells silenced for Merlin showed increased levels of the OPN transcript and OPN promoter (luciferase) activity indicating significantly (p < 0.05) upregulated transcription of OPN (Figure 3A & 3B). In contrast, SUM159 cells restored for Merlin showed notably (p < 0.05) reduced activity of the OPN promoter (Figure 3C). In the perspective of our results indicating that Merlin modulates β-catenin signaling, we analyzed the sequence of the human OPN promoter and discovered three putative TCF/LEF-recognition sites within the OPN promoter. We generated distinct reporter constructs, each containing one binding site repeated three times in tandem upstream of the luciferase reporter - JS (3x CACAAG), AS (3x GAAAAAG), and KS (3x AACAAG). SUM159 cells were co-transfected with each of the three luciferase reporter constructs and either empty vector or a Merlin-expressing construct. Co-transfection of Merlin significantly suppressed the activity of all three luciferase constructs. Furthermore, co-transfection of Merlin resulted in the greatest suppression of the most 3′ construct, JS (Figure 3D). As such, it appears that all three sites are regulated by Merlin-induced reduction in β-catenin activity, with the 3′ JS site (CACAAG) having the most impact. In order to ascertain that this putative TCF/LEF binding site within the OPN promoter binds β-catenin, we conducted a ChIP analysis with anti-β-catenin or anti-IgG followed by PCR amplification of the OPN promoter region that encompasses the JS TCF/LEF-binding site. As seen in Figure 3E, PCR following ChIP with β-catenin yielded a robust band with JS TCF/LEF-site-specific primers relative to non-specific primers that did not harbor a putative TCF/LEF binding site (−2693 to −3225bp, 950bp upstream of the transcription start site). This is evident by assessment of the product by quantitative PCR, showing 12-fold increase in the product abundance when immunoprecipitated with JS-specific primers (Figure 3E). These data collectively suggest that Merlin regulates OPN by inhibiting β-catenin associated TCF/LEF transcription of OPN.

### Merlin reduces β-catenin protein levels by targeting β-catenin for proteasomal degradation

**Figure 4:**
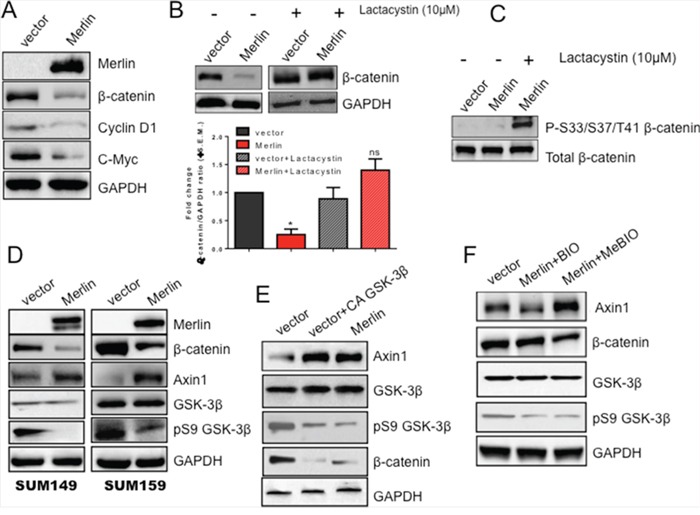
Merlin increases GSK-3β-mediated upregulation of Axin1 protein levels leading to proteasomal degradation of β-catenin protein **A.** Merlin reduces β-catenin protein levels as well as downstream targets cyclin D1 and c-Myc. Lysates from SUM159 cells stably expressing Merlin were immunoblotted with anti-Merlin, anti-β-catenin, anti-cyclin D1, and anti-c-Myc antibodies. GAPDH served as a loading control. **B.** β-catenin expression is restored upon treatment with the proteasome inhibitor lactacystin. SUM159 Merlin-expressing cells and the corresponding control cells were treated with 10μM lactacystin for 16 hours. Lysates were immunoblotted with anti-β-catenin antibody. GAPDH was used as a loading control. Bar graph represents densitometric analysis completed using GeneTools (Syngene) software comparing band intensities of lane 2 with lane 1; lane 4 compared with lane 3. Bars represent β-catenin divided by GAPDH. These blots were repeated once. Merlin significantly reduces β-catenin (p=0.001); proteasome inhibition restores β-catenin protein levels (ns=not significant). * indicates statistically significant differences relative to respective control. **C.** Degradation-targeted β-catenin is arrested and apparent in Merlin-expressing cells upon proteasomal inhibition. SUM159 cells stably expressing Merlin were treated with 10μM lactacystin for 18 hours. Lysates were evaluated by western blot analysis. Total β-catenin served as a loading control. **D.** Merlin increases Axin1 protein levels concomitant with decreased β-catenin protein levels in SUM149 and SUM159 breast cancer cells. Cells transfected with empty pcDNA3.1 vector or pcDNA3.1-Merlin were lysed and analyzed by immunoblotting. GAPDH serves as a loading control for all groups. **E.** A constitutively active (CA) GSK-3β mutant restores Axin1 levels. SUM159 cells stably expressing empty pcDNA3.1 vector were transfected with CA GSK-3β. Thirty-six hours post-transfection, cell lysates were immunoblotted for the indicated molecules. GAPDH served as a loading control. **F.** Inhibition of GSK-3β decreases Axin1 protein levels while restoring β-catenin protein levels. SUM159 cells stably expressing Merlin were treated with 0.5μM GSK-3β Inhibitor X (BIO) or its corresponding control (MeBIO) for 18 hours. Cell lysates were assessed by western blotting. GAPDH served as a loading control.

Our evidence suggests that loss of Merlin causes re-localization of β-catenin and decreases its transcriptional activity. The increased time for visualizing β-catenin in cells restored for Merlin suggested probable changes in cellular β-catenin levels. Therefore we evaluated the levels of total β-catenin from the perspective of Merlin. The total cellular level of β-catenin is decreased in SUM159 cells restored for Merlin expression, simultaneous with a decrease in the transcriptional targets, cyclin D1 and c-Myc (Figure 4A). There was no appreciable decrease in the steady state levels of β-catenin transcript (Supplementary Data 2A). To address the role of proteasomal degradation of β-catenin in reducing its protein abundance, we treated SUM159 cells stably expressing empty vector or Merlin with the proteasome inhibitor clasto-lactacystin β-lactone (Lactacystin). Upon proteasome inhibition, β-catenin levels were restored even in the presence of Merlin (Figure 4B). To further test this, we treated SUM159 cells restored for Merlin with lactacystin and assessed the expression of degradation-targeted β-catenin. Degradation-targeted β-catenin is phosphorylated at key residues (S33/S37/T41) that mark this protein for proteasome-mediated elimination. When normalized to total β-catenin, there was an increase in the accumulation of phosphorylated/degradation-targeted β-catenin in lactacystin-treated cells (Figure 4C). Taken together these data suggest that not only does Merlin inhibit the Wnt/β-catenin pathway by altering β-catenin's sub-cellular localization, but Merlin also decreases overall levels of β-catenin protein.

### Merlin decreases β-catenin protein levels by upregulating the destruction complex protein Axin1

Since we observed that Merlin decreases β-catenin protein levels, we aimed to determine the mechanism regulating this decrease. In the absence of active Wnt signaling, β-catenin is phosphorylated by casein kinase Iα (CKIα) at Serine 45 [37-39] and by glycogen synthase kinase-3β (GSK-3β) at Serine residues 33 and 37 and Threonine 41 [40]. These phosphorylation events are made possible by the scaffolding proteins Axin and APC bringing β-catenin in close contact with CKIα and GSK-3β. Together, these proteins (Axin, APC, CKIα and GSK-3β) comprise the destruction complex that targets β-catenin for ubiquitination by the E3 ubiquitin ligase β-TrCP and subsequent proteasomal degradation [41-43]. A recent report documented that Axin1 expression is decreased in breast cancer [29]. Axin1 inactivation results in stabilization of Myc, a downstream target of Wnt signaling [44]. As such, we sought to determine the effect of Merlin on Axin1. While the transcript levels of Axin 1 are comparable (Supplementary Data 2B), we observed an increase in Axin1 protein levels in both SUM149 and SUM159 metastatic breast cancer cell lines restored for Merlin concomitant with a decrease in β-catenin protein levels. This increase in Axin1 also correlated with decreased levels of inactive GSK-3β (Figure 4D). Wnt stimulation inhibits phosphorylation of Axin1 by GSK-3β, leading to a release of β-catenin from the destruction complex, culminating in increased β-catenin-mediated activity [45]. As such, we assessed the impact of restoring GSK-3β activity on Axin1 expression by transfecting cells with a constitutively active (CA) form of GSK-3β. Upon introduction of CA-GSK-3β, Axin1 protein was restored to levels comparable with that in SUM159 Merlin-expressing cells (Figure 4E). To be thorough in our analysis of this relationship, we inhibited GSK-3β activity in SUM159 Merlin-expressing cells by treating cells with a GSK-3β inhibitor (BIO), as well as its corresponding inactive control (MeBIO). BIO-mediated GSK-3β inhibition of SUM159 Merlin-expressing cells resulted in reduced Axin1 levels concomitant with the restoration of β-catenin (comparable to vector control cells) (Figure 4F). Axin1 levels were reduced by 30% compared to vector control cells and 50% when compared to the MeBIO-treated cells. These results collectively suggest that Merlin upregulates Axin1 protein levels mediated by GSK-3β phosphorylation leading to proteasomal degradation of β-catenin and consequently reduced activity.

### Degradation-resistant β-catenin restores malignant activity in Merlin-expressing breast cancer cells

**Figure 5:**
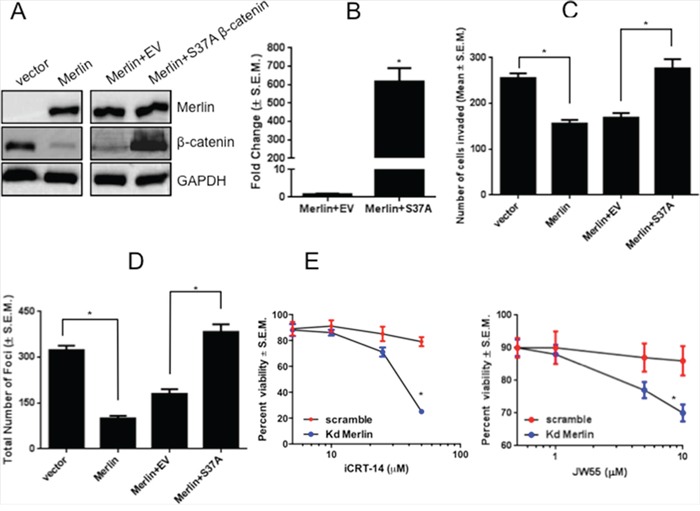
Expression of a degradation-resistant β-catenin mutant rescues malignant activity of breast cancer cells in presence of Merlin **A.** Expression of degradation-resistant β-catenin in SUM159 Merlin-expressing breast cancer cells. SUM159 cells stably expressing Merlin were transfected with empty pcDNA3.1 vector or pcDNA3.1-S37A β-catenin. Twenty-four hours post-transfection, cell lysates were subjected to immunoblotting for β-catenin and Merlin. GAPDH served as loading control. Transfection with S37A β-catenin results in β-catenin expression in the presence of Merlin (lane 4 vs. lanes 2-3). **B.** Expression of degradation-resistant β-catenin promotes TOPFlash activity in presence of Merlin (p < 0.0001). SUM159 cells stably expressing Merlin were co-transfected with degradation-resistant S37A β-catenin mutant (200 ng/well) and TOPFlash vector (100 ng/well). Luciferase activity was assessed after 30-33 hours and normalized to total protein concentration. Fold change in activity was calculated with vector control (EV) set to 1. * indicates statistically significant differences relative to respective control. **C.** Expression of degradation-resistant β-catenin restores invasive capabilities in presence of Merlin. SUM159 Merlin-expressing cells were transfected with empty pcDNA3.1 vector or pcDNA3.1-S37A β-catenin and were seeded in the upper chamber of Matrigel-coated inserts with 8 μm pores. The lower chamber was filled with medium containing 10μg/mL fibronectin. Cells were incubated for 10 hours, and stained with crystal violet solution. Eight random fields were photographed. Cells that invaded through the filter were counted using the Nikon Elements software package. Merlin significantly suppresses invasion (p=0.0015), whereas the degradation-resistant β-catenin restores invasive capabilities in presence of Merlin (p=0.008). * indicates statistically significant differences relative to respective control. **D.** Expression of S37A-β-catenin restores contact-independent growth capabilities in presence of Merlin. Colonies with >50 cells were manually counted after staining with crystal violet. The assay was repeated twice and the graph is representative of the result. Merlin significantly reduces foci formation (p=0.0002) and S37A-β-catenin restores contact-independent growth capabilities in presence of Merlin (p=0.0003). * indicates statistically significant differences relative to respective control. **E.** Merlin sensitizes cells to inhibitors of Wnt/β-catenin signaling. MCF10AT cells stably silenced for Merlin were plated at a density of 5,000 cells per well in 96 well tissue culture plates and treated with Wnt/β-catenin inhibitors iCRT-14 and JW55. Cell viability was scored by MTS assay.

We previously determined that Merlin inhibits tumor growth and invasive characteristics in breast cancer cells [13]. To assess whether this effect is mediated by decreased β-catenin protein levels, we transfected SUM159 cells restored for Merlin with a degradation-resistant β-catenin mutant (S37A). Phosphorylation of β-catenin at Serine37 by GSK-3β targets β-catenin for degradation. The S37A mutant mimics the de-phosphorylated, stable form of β-catenin and persists in cells in the presence of Merlin (Figure 5A). The degradation-resistant S37A β-catenin was able to significantly (p < 0.05) upregulate TOPFlash activity even in the presence of Merlin (Figure 5B). While Merlin restricts tumor invasive abilities (Figure 5C; Supplementary Data 2C), the S37A β-catenin mutant restored invasive capabilities of SUM159 breast cancer cells stably-expressing Merlin (Figure 5C) and enabled these breast cancer cells to escape contact-inhibition of growth (Figure 5D). These results suggest that Merlin's function as a suppressor of malignant activity of tumor cells is mediated by proteasomal degradation and subsequently reduced levels of β-catenin protein.

Given our findings that cells compromised for Merlin expression show robust β-catenin-mediated signaling, we tested the sensitivity of Merlin-silenced MCF10AT breast cancer cells to two inhibitors of Wnt/β-catenin signaling. We tested iCRT-14, an inhibitor of β-catenin-responsive transcription and JW55, an inhibitor of the PARP domain of tankyrase 1 and tankyrase 2 (TNKS1/2), regulators of the β-catenin destruction complex. By virtue of inhibiting TNKS1/2 poly(ADP-ribosyl)ation activity, JW55 stabilizes AXIN2, leading to increased degradation of β-catenin. Our findings revealed that cells silenced for Merlin and having greater activity of β-catenin were sensitive to iCRT-14 and JW55 Wnt/β-catenin signaling inhibitors (Figure 5E). As such, our data clearly suggests that breast cancer cells with reduced Merlin expression are sensitive to compounds that inhibit β-catenin.

## DISCUSSION

Functionally, Merlin is best characterized for its role in contact inhibition of growth. Merlin is directly involved in cytoskeletal organization via its ability to bind actin and tubulin and inhibits the actin nucleation promotion factor N-WASP, thereby regulating actin polymerization [46]. Merlin also binds adhesion molecules including Δ1 integrin and layillin and also molecules such as paxillin and focal adhesion kinase which are part of the focal adhesion complexes [47-49]. Merlin-deficient cells display lack of contact inhibition and demonstrate cellular protrusions that are actin-rich [50]. Merlin-deficient cells have impaired cell-cell contact due to destabilized adherens junctions and display aberrant adhesion to the extracellular matrix [31]. We previously reported that Merlin is regulated at the post-translational level by outside-in signaling in breast cancer. The loss of Merlin seen in advanced breast cancer tissues was largely due to Merlin's proteasomal degradation and not due to mutations or reduced transcript levels [13, 51]. A recent genome-wide *in vivo* CRISPR-Cas9 screen in mice revealed a set of genes regulating lung metastasis. In particular, the *Nf2* gene locus was found to be significantly enriched in late primary tumor and lung metastases suggesting that Merlin likely plays an important role in promoting metastasis as well as the growth of the primary tumor [52].

At the cell membrane, β-catenin associates with E-cadherin and α-catenin in a dynamic complex and is an integral component of the adherens junction. Dismantling of this complex is associated with the release of β-catenin and concomitant loss of cell-cell contact that can facilitate cell migration/motility. Loss of membranous β-catenin and the resulting cytosolic/nuclear accumulation of this protein is a significant event frequently observed in human breast cancers [53-55]. It is postulated that this event occurs at a later stage of tumor progression and signifies higher tumor grade [56] and poor prognosis [57]. As such, here we queried the possibility that loss of Merlin modulates tumor cell malignancy by impacting β-catenin. Not only do Merlin and β-catenin interact, but the loss of Merlin appears to untether β-catenin from the membrane resulting in a diffuse or nuclear re-location. This was concomitant with an increase in β-catenin-mediated transcriptional activity. Thus, Merlin is critical for membranous β-catenin localization. Furthermore, breast and pancreatic cancer cells restored for Merlin showed a decreased cellular pool of β-catenin. We uncovered that Merlin upregulates the destruction complex-associated protein Axin1, initiated by GSK-3β activation. Axin1, recently determined to be a critical protein that is downregulated in breast cancer [29], targets β-catenin for degradation when complexed with APC, GSK-3β, and CKII. Upon GSK-3β inhibition, Axin1 levels in Merlin-expressing cells are reduced concomitant with an increase in β-catenin protein levels, suggesting that Merlin's effect on β-catenin is mediated by GSK-3β-initiated upregulation of Axin1. This is in agreement with previous studies that cumulatively report that GSK3β regulates Axin1 facilitating its interaction with β-catenin [58, 59]. Aberrant activation of β-catenin-mediated signaling was characterized by the upregulation of its *bonafide* targets c-Myc, cyclin D1, and OPN. In support of this observation, MCF7 cells silenced for Merlin registered a robust increase in the level of β-catenin associated with the TCF4 transcription factor (Supplementary Data 2C). Using detailed molecular dissection approaches, we were able to conclusively attribute a role for β-catenin in transcriptionally upregulating OPN in human cancer. The oncogenic and tumor-promoting properties of OPN have been well-recognized. In multiple investigations, including ours, OPN has been demonstrated to increase the metastatic properties of cancer cells, with the general consensus being that OPN levels are elevated in cancer and associated with worse prognosis in breast and pancreatic cancer [60-69].

Our findings bear exciting implications from multiple perspectives. With the identification of upregulation of OPN upon the loss of Merlin, the association between Merlin and OPN becomes reciprocal. Our group was the first to report that OPN-mediated signaling causes Merlin to be phosphorylated at S315, marking it for proteasome-mediated degradation [13, 51]. With the current study in perspective, it is apparent that microenvironment-derived signaling encompassing OPN causes a decrease in the cellular pools of Merlin; in turn the unrestricted activation of β-catenin increases the levels of OPN. This vicious feed-forward relationship between Merlin and OPN further keeps a check on Merlin levels in the tumor cells, enabling tumor progression (Supplementary Data 2D). Importantly, our study also uncovers, for the first time, that Merlin is important in pancreatic cancer. In particular, we determined that loss of Merlin in pancreatic cancer cells increases the transcriptional activity of β-catenin. In light of recent literature documenting an important role for Wnt/β-catenin signaling in pancreatic carcinoma [70-72], our findings have profound implications. Cumulatively, our findings suggest that by virtue of thwarting β-catenin's growth-promoting activity, Merlin keeps a check on malignant progression of cancer. A homozygous knockout of *Nf2* in mice is embryonic lethal with the mice failing to gastrulate between days 6.5 and 7 [73]; this indicates that Merlin is a critical molecule in mammalian embryonic development. This coincides with the organized re-location of β-catenin in the primitive streak and mature node of the developing mouse embryo [74]. Thus, the crosstalk between Merlin and Wnt/β-catenin signaling likely begins early in development and is invoked once again during tumor progression and malignancy.

Our work also has drawn upon the possibility of therapeutically targeting the aberrantly activated β-catenin. We used two inhibitors of the Wnt/β-catenin pathway that act at distinct levels. iCRT-14 inhibits β-catenin–mediated transcription and JW55, a tankyrase inhibitor that stabilizes Axin and leads to the rapid destruction of β-catenin. Given our findings that the loss of Merlin compromises the levels of Axin1 simultaneous with aberrant activation of β-catenin, both these inhibitors show increased effectiveness in eliminating cells with upregulated β-catenin resulting from the loss of Merlin. As such, we have identified a putative therapeutic approach for tumor cells that have compromised Merlin expression with concomitant upregulation of β-catenin. Despite the importance of the Wnt pathway in breast and pancreatic cancer, there are surprisingly few therapies that target this pathway with even fewer that have yielded positive results. Inhibitors of this pathway are now being evaluated in clinical trials. At the time of preparation of this manuscript, Porcupine inhibitor LGK-974 (Novartis) [75] and β-catenin activity inhibitor PRI-724 (Prism Pharma) are in trials for advanced solid and hematological malignancies. In the perspective of these advances, our findings are timely and strongly plant Merlin as a vital determinant of malignant attributes of breast and pancreatic cancers and provide evidence for Merlin in restraining aberrant activation of Wnt/β-catenin signaling.

## MATERIALS AND METHODS

### Cell culture

SUM159 and SUM149 cells were grown in DMEM/F-12 supplemented with heat-inactivated FBS (Atlanta Biologicals, Atlanta, GA), insulin, and hydrocortisone. MCF7 cells were cultured in DMEM/F-12 supplemented with FBS and insulin. MCF10AT cells were cultured as described previously [13]. Stable Merlin-expressing and silenced transfectants were cultured in media supplemented with G418 or puromycin. BxPC-3, HPAF-II, and MIA PaCa-2 cells were cultured in DMEM/F12 supplemented with 10% heat-inactivated FBS. Capan-1 cells were cultured in DMEM/F12 supplemented with 20% heat-inactivated FBS. SUIT-2 cells were cultured in ATCC formulation RPMI supplemented with 10% heat-inactivated FBS. Panc 10.05 cells were cultured in the same supplemented with 15% FBS and insulin. SUIT-2 cells were cultured in RPMI 1640 with 10% FBS. Cell lines were obtained from ATCC and Asterand, Inc. They were routinely tested and confirmed to be free of Mycoplasma.

Stable Merlin-expressing transfectants of BxPC-3 cells were generated by transfecting pcDNA3-Merlin or an empty-vector (pcDNA3) as previously described [13] and were selected in 250 μg/ml G418 (Invitrogen, Carlsbad, CA). Stable Merlin-silenced MIA Paca-2 and SUIT-2 cells were generated by transfecting Merlin-knockdown construct pLenti-H1-Si631-NF2-U6-CMV-GFP-2A-Puro (Capital Biosciences, MD). Scrambled construct pLenti-H1-SCR-U6-CMV-GFP-2A-Puro was transfected as control. Stable Merlin-knockdown transfectants of MIA PaCa-2 and SUIT-2 were selected in 350ng/ml puromycin and 1.25μg/ml puromycin (Calbiochem, MA), respectively. All cells were cultured in a humidified 5% CO_2_ incubator at 37°C.

### Cell proliferation assay

Cells were plated at a density of 5,000 cells per well in 96 well tissue culture plates and treated with Wnt/β-catenin inhibitors iCRT-14 (Tocris Bioscience, Minneapolis, MN, USA) or JW55 (Tocris Bioscience) or vehicle control (DMSO). Cell viability was determined after 72 hours using the PMS/MTS reagent (Promega Corporation, Madison, WI). Absorbance was read at 490 nm.

### Plasmids and constructs

M50 TOPFlash luciferase construct was generously gifted by Dr. Randall Moon (University of Washington, Seattle, WA). pACT-Merlin was generated using the following primers: Forward primer 5′(GCACTAGTCTAGAGCCGGGGCCATCGCTTC)3′ and reverse primer 5′(CGTCGGGGTACCGAGCTCTTCAAAGAAGGCCA)3′. pcDNA3.1-Merlin was used as a starting template (gifted by Dr. David Gutmann, Washington University, St. Louis, MO). pBIND-β-catenin was generated using the forward primer: 5′(GCACTAGTCTAGAGCTACTCAAGCTGATTTGATG)3′ and reverse primer 5′(CGTCGGGGTACCCAGGTCAGTATCAAACCAGG)3′. pcDNA3.1-β-catenin was used as a template. pcDNA3.1-β-catenin and the S37A degradation-resistant plasmids were generated in the lab.

### Luciferase reporter assays

#### TOPFlash assay

Cells were co-transfected with 200 ng/well each of β-galactosidase and TOPFlash vector and the effector plasmids using Lipofectamine 2000 (Invitrogen) as per the manufacturer's instructions. Each group was assayed in triplicate with luciferase readings being normalized to β-galactosidase. Data are expressed as relative luciferase activity, where control is 100%. For the TOPFlash assay with degradation-resistant β-catenin, the same basic procedure was followed as described above. Cells were co-transfected with 200 ng/well empty pcDNA3.1 vector or pcDNA3.1 S37A β-catenin as the effector and 100 ng/well TOPFlash construct. After 36 hours, luciferase activity was measured using the Luciferase Assay System (Promega) and normalized to total protein concentration.

#### Mammalian two-hybrid assay

pACT-Merlin (50ng/well), pBIND-β-catenin (50ng/well), and pGL4.31 (100ng/well) were co-transfected in SUM159 cells using Lipofectamine™ 2000 (Invitrogen) as per manufacturer's instructions. Total protein was harvested and luciferase activity was measured.

#### OPN promoter assay

OPN promoter (200ng) [76] was co-transfected with either Merlin siRNA or shRNA in MCF10AT cells. In SUM159 cells, the OPN promoter was co-transfected with the Merlin-expressing construct or a corresponding control vector. The luciferase reading was normalized to the total protein concentration. Data are expressed as fold change in luciferase activity where control is the baseline. The experiments were repeated three times with the graph being representative of the results.

### Co-immunoprecipitation

SUM159 cells transfected with Merlin were lysed and 1 mg of lysate was immunoprecipitated with anti-Merlin or anti-β-catenin as a reverse co-IP using the following protocol: Primary antibody was added to the lysate at a 1:50 dilution and samples were incubated at 4°C for 2 hours with gentle rocking. Protein A/G agarose beads (sc-2003, Santa Cruz Biotechnology, Santa Cruz, CA) were added and the samples were returned to 4°C for rocking overnight. Samples were centrifuged and pellet was washed with ice-cold PBS. Pellet was re-suspended in sample buffer, vortexed and centrifuged at maximum speed for 30 seconds at room temperature. Samples were resolved on a 10% SDS PAGE gel. The immunoprecipitates were assessed by immunoblotting. The interaction between TCF4 and β-catenin in MCF7 cells was assessed by immunoprecipitating the lysate with with anti-TCF4 (L40C3; #2953, Cell Signaling) followed by immunoblotting for β-catenin.

### Immunofluorescence

SUM159 and MCF7 transfectants were fixed using 4% paraformaldehyde. Following two washes with ice-cold PBS, cells were permeabilized in PBS containing 0.1% Triton X-100 (PBST) and incubated with 1% BSA in PBST to block non-specific binding of the antibodies, followed by incubation with anti-Merlin (clone E-2, Santa Cruz) and anti-β-catenin (#9582, Cell Signaling, Danvers, MA or #610156, BD Transductions, Franklin Lakes, NJ) antibodies. Following three washes with PBS, cells were incubated with the mixture of two secondary antibodies each at 1:100 dilution (TRITC-conjugated mouse and FITC-conjugated rabbit, Sigma, St. Louis, MO) in 1% BSA in the dark. Cells were washed three times in PBS and mounted in DAPI (Vectashield, Vector Laboratories, Burlingame, CA). Cells were visualized using a Nikon Eclipse TE2000-U microscope and photographed at 60X magnification using the F3.8 setting of a Nikon CoolPixId700D camera.

### Western blotting analysis

Immunoblotting was performed with the following antibodies from Cell Signaling Technologies (Danvers, MA): anti-β-catenin, anti-pS552-β-catenin, anti-pS675-β-catenin, anti-pS33/S37/T41-β-catenin, anti-Axin1, anti-TCF4, and anti-GAPDH. Anti-Merlin antibodies were purchased from Santa Cruz Biotechnology (Santa Cruz, CA). Anti-c-Myc antibody was purchased from Clontech. Anti-active β-catenin antibody was purchased from Millipore. Anti-rabbit or anti-mouse HRP conjugated secondary antibody was used for detection and blots were developed with SuperSignal substrate (Pierce, Rockford, IL) and exposed using a Syngene G:Box Imager.

### Size exclusion chromatography [fast protein liquid chromatography (FPLC)]

MCF10AT cells were grown to ~70 % confluence; cells were lysed in NP-40 lysis buffer and fractionated using a Superose 6 HR 10/30 size exclusion column (Amersham Biosciences, Piscataway, NJ) on a BIO-RAD BioLogic DuoFlow coupled with a BioLogic Fraction Collector. The column was run using PBS at a flow rate of 0.25 ml/min. Fractions (1ml) were collected and subjected to immunoblotting. Fractions 14, 15, & 16 were pooled and immunoprecipitated with anti-Merlin or anti-β-catenin using the protocol outlined in co-immunoprecipitation section. The immunoprecipitates were evaluated by immunoblotting.

### Chromatin immunoprecipitation (ChIP) assay

Following the manufacturer's instructions for the CHIP-IT™ Express Enzymatic Kit (Active Motif, Carlsbad, CA), SUM159 cells were cultured to 70-80% confluence and the cells were fixed (0.54 mL 37% formaldehyde + 20 mL DMEM/F-12) for 10 minutes at room temperature. Cells were scraped and transferred to an ice-cold dounce homogenizer and homogenized on ice 20 times to release the nuclei and centrifuged at 5,000 rpm for 10 minutes at 4°C. The nuclear pellet was re-suspended in the kit provided Digestion Buffer and the DNA was sheared enzymatically following the manufacturer's directions and immunoprecipitated for β-catenin. GAPDH primers were provided in the kit (Active Motif). The immunoprecipitate was subjected to PCR using primers specific to JS and non-specific primers. The non-specific primers (IDT Coralville, Iowa) are as follows: Forward (5′ GCAACTTTATAATCTGTGTGC 3′); Reverse (5′ GGTAGGGGGAAATATGTCTC 3′). The JS primers (Operon, Huntsville, Alabama) are as follows: Forward (5′ TGTCACTAGTGCCATTTGTCT 3′); Reverse (5′ CTCTTGCCTGTATGATTGTACT 3′). PCR products were visualized on a UVP (Upland, CA) transilluminator under ultraviolet light.

Following DNA isolation in the final step of the ChIP, quantitative PCR was done to quantify the extent of specific pull-down. The negative Human Control Primer Set supplied in the ChIP kit was used as endorse control and the fold change of expression was calculated using the Isotype Antibody Control as the reference set.

### Invasion and foci formation assays

Invasion assay was accomplished using 8μM polyethylene terpthalate filters (BioCoat™ Matrigel Invasion Chambers, BD Pharmingen), as described earlier [13]. Cells were allowed to invade through Matrigel coated filters for 16 hours. The cells invaded to the lower sides of the transwell were fixed using 4% (w/v) paraformaldehyde and stained using 0.05% crystal violet. Each test group was assayed in triplicate and the assay repeated at least twice. For foci formation analysis, cells were seeded sparsely (500 cells in a 10cm culture dish) in medium containing selection antibiotics. Foci formed were counted after 10–14 days.

### Statistical analyses

Statistical differences between groups were assessed using the student's t-test or ANOVA, using GraphPad Prism 5 software. Statistical significance was determined if the analysis reached 95% confidence. The precise p-values are listed in the corresponding Figure Legends.

## SUPPLEMENTARY FIGURES


