# Supplementary material for: Loss of tumor suppressor Merlin results in aberrant activation of Wnt/β-catenin signaling in cancer
Source: Oncotarget. 2016 Feb 19;7(14):17991–8005. doi: 10.18632/oncotarget.7494 (PMC4951266; doi:10.18632/oncotarget.7494)
Supplement: Supplementary file 1 [file oncotarget-07-17991-s001.pdf]

SUPPLEMENTARY FIGURES

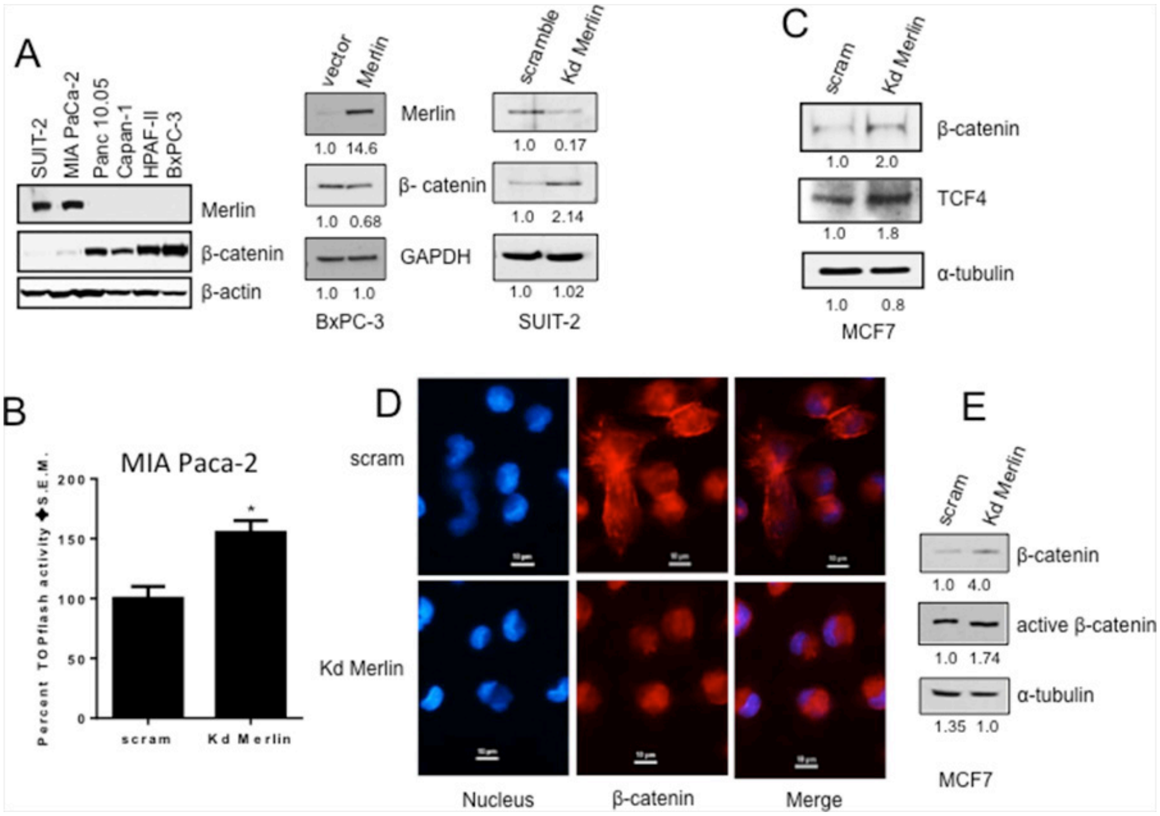

Supplementary Data 1: Pancreatic cancer cell lines were analyzed for the level of Merlin and total β-catenin by Western blot analysis. A. β-actin, GAPDH or alpha-tubulin served as a loading control. Also shown are western blots validating that exogenous expression of Merlin in pancreatic cancer BxPC-3 causes a decrease in the protein levels of β-catenin. Silencing endogenous Merlin in SUIT-2 pancreatic cancer cells causes an increase in the protein levels of β-catenin. **B.** Silencing of endogenous Merlin in MIA PaCa-2 cells results in significant upregulation ($p=0.024$) of TOPFlash activity. TOPFlash construct was co-transfected with a scrambled construct or shRNA construct (Kd Merlin). * indicates statistically significant differences relative to respective control. **C.** Abrogating Merlin expression in MCF7 cells enables robust interaction between β-catenin and TCF4 relative to the scrambled-control cells. Cell lysate was immunoprecipitated for TCF4 and immunoblotted for β-catenin. α-tubulin serves to normalize the blots. The numbers below each panel indicate the relative densitometric intensities. **D.** MIA PaCa-2 cells transfected with a scrambled control construct or a construct expressing shRNA for Merlin (Kd Merlin) were stained for β-catenin and mounted in DAPI (Vectashield). Loss of Merlin causes β-catenin to relocalize from a predominantly membranous presence to a diffuse cellular/nuclear presence. **E.** The levels of active β-catenin are increased in MCF7 cells upon silencing endogenous Merlin. α-tubulin serves as a loading control. Cell lysates were immunoblotted; the intensity of the bands was quantitated by densitometry. The numbers below each band depict the relative band intensity.

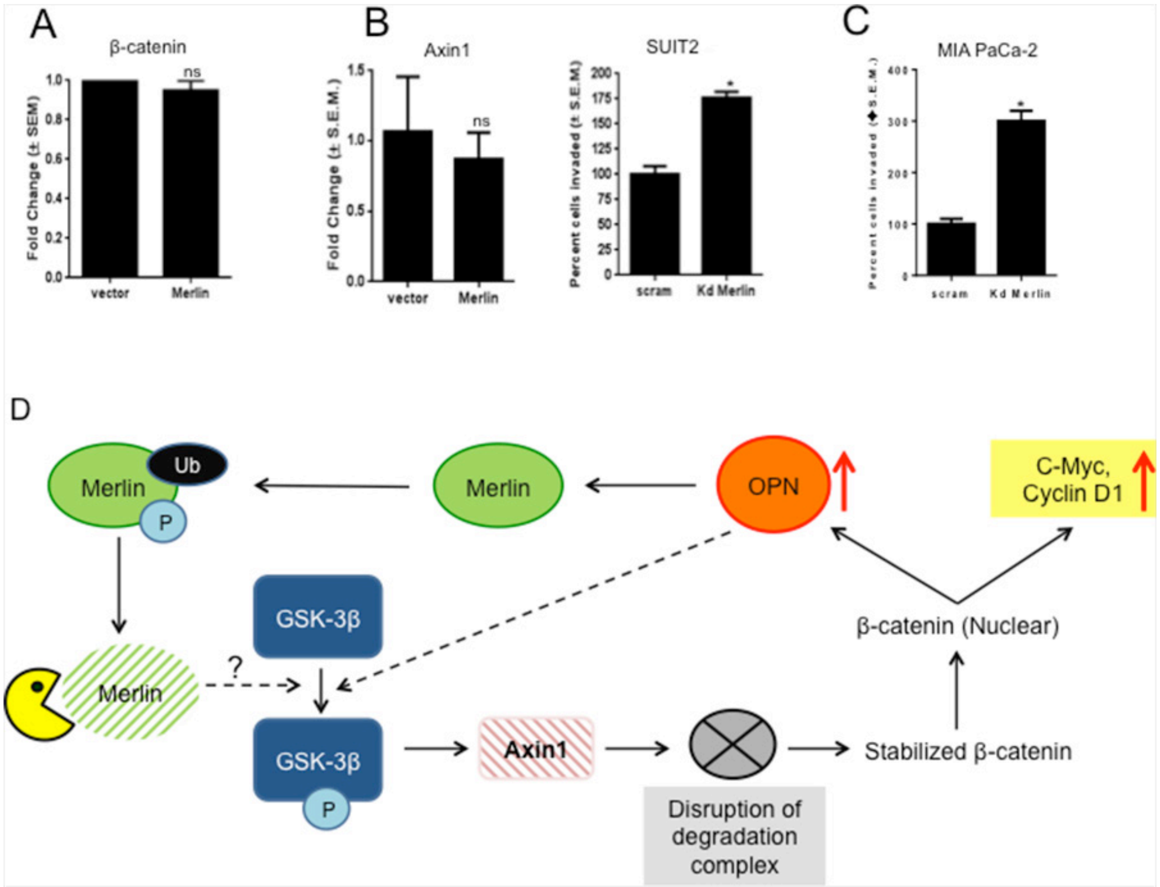

Supplementary Data 2: A. Transcript levels of β-catenin are unaltered in SUM159 cells transfected for Merlin expression (ns=not significant). Transcript levels were assessed by quantitative real-time RT-PCR. GAPDH was used as an internal control. **B.** Axin1 transcript levels are comparable between SUM159 vector control and Merlin-expressing cells (ns=not significant). Transcript levels were assessed by quantitative real-time RT-PCR. GAPDH was used as an internal control. **C.** Silencing endogenous Merlin in SUIT-2 and MIA PaCa-2 cells caused a significant increase in the numbers of cells invaded through Matrigel (p=0.04 for SUIT-2 and p=0.0009 for MIA PaCa-2). * indicates statistically significant differences relative to respective control. **D.** Merlin's proposed mechanism of action. Loss of Merlin enables the nuclear translocation of β-catenin through a mechanism involving inactivation of GSK3β and reduced Axin1 levels. Reduced Axin1 compromises the formation of the destruction complex, facilitating stabilization and nuclear translocation of β-catenin. OPN represents a vital transcriptional target of β-catenin that can mark Merlin for proteasome-mediated elimination. Cyclin D1 and c-Myc are also transcriptionally upregulated by β-catenin and promote proliferation. Thus, there is a vicious feed-forward loop that ensures elimination of Merlin. The dotted line indicates possible mechanisms that can regulate the inactivation (phosphorylation) of GSK-3β (? indicates not characterized thus far).
